# Crystal structure of the spliceosomal DEAH-box ATPase Prp2

**DOI:** 10.1107/S2059798318006356

**Published:** 2018-06-08

**Authors:** Andreas Schmitt, Florian Hamann, Piotr Neumann, Ralf Ficner

**Affiliations:** a Department of Molecular Structural Biology, Institute of Microbiology and Genetics, GZMB, Georg-August-University Göttingen, Justus-von-Liebig-Weg 11, 37077 Göttingen, Germany

**Keywords:** spliceosome, RNA helicase, DEAH-box, Prp2

## Abstract

The DEAH-box ATPase Prp2 plays a key role in the activation of the spliceosome as it promotes the transition from the B^act^ to the catalytically active B* spliceosome. Here, four crystal structures of Prp2 are reported: one of the nucleotide-free state and three different structures of the ADP-bound state. The overall conformation of the helicase core, formed by two RecA-like domains, does not differ significantly between the ADP-bound and the nucleotide-free states. However, intrinsic flexibility of Prp2 is observed, varying the position of the C-terminal domains with respect to the RecA domains. Additionally, in one of the structures a unique ADP conformation is found which has not been observed in any other DEAH-box, DEAD-box or NS3/NPH-II helicase.

## Abstract 2

The DEAH-box ATPase Prp2 is a key player during pre-mRNA splicing. Here, the first four crystal structures of this spliceosomal factor are reported in two different states, and the flexibility of its C-terminal domains and a previously undescribed ADP conformation are reported.

## 1. Introduction   

In eukaryotic cells, precursor messenger RNAs (pre-mRNAs) may contain noncoding intervening sequences (introns) that have to be removed by splicing prior to nuclear export and translation of the mRNAs. The process of pre-mRNA splicing is carried out by a highly dynamic multi-megadalton ribo­nucleoprotein (RNP) complex, the spliceosome, which catalyzes two sequential transesterification reactions (Will & Lührmann, 2011 ▸; Hoskins & Moore, 2012 ▸; Matera & Wang, 2014 ▸). For each intron to be excised, the spliceosome assembles stepwise onto the pre-mRNA from five small nuclear ribonucleoprotein particles (snRNPs), named U1, U2, U4/U6 and U5, and a large number of non-snRNP proteins (Wahl *et al.*, 2009 ▸). Briefly, the assembly starts with the U1 snRNP binding to the 5′ splice site of the pre-mRNA and the U2 snRNP interacting with the branch-point sequence (BPS), resulting in the formation of the spliceosomal A complex. Subsequently, the pre-assembled tri-snRNP consisting of the U4/U6 and U5 snRNPs is recruited to the spliceosome, leading to the formation of the catalytically inactive B^act^ complex. In the subsequent step of the splicing cycle, the catalytically active B* complex is formed which facilitates the first transesterification reaction. After this, complex C is made by further remodelling and the second transesterification reaction takes place. Finally, the spliceosome is disassembled, leading to release of the spliced mRNA and the intron lariat.

During assembly, the splicing reaction and disassembly, the spliceosome undergoes large compositional and conformational changes, including the remodelling of RNA–RNA, RNA–protein and protein–protein interactions (Wahl *et al.*, 2009 ▸). These rearrangements are mainly driven by eight DE*x*D/H-box ATPases which use the energy from ATP hydrolysis to unwind dsRNA and/or remodel RNA–protein interactions (Cordin *et al.*, 2012 ▸; Ding & Pyle, 2012 ▸; Ozgur *et al.*, 2015 ▸). These helicases belong to helicase superfamily 2 (SF2) and share a common fold consisting of two RecA-like motifs which together form the helicase core (Fairman-Williams *et al.*, 2010 ▸). In DE*x*D/H-box helicases, the RecA domains harbour eight conserved sequence motifs named I, Ia, Ib, II, III, IV, V and VI, which are involved in RNA and/or nucleotide binding, with motif II carrying the name-giving consensus sequence DE*x*D/H (Cordin *et al.*, 2006 ▸). Additional domains located C-terminal to the helicase core have been shown to act as a binding platform for interaction partners or have a regulatory effect on the ATPase activity of the helicase (Cordin & Beggs, 2013 ▸; Kudlinzki *et al.*, 2012 ▸).

The transition to the catalytically active B* complex is promoted by the DEAH-box ATPase Prp2 together with its cofactor Spp2 (King & Beggs, 1990 ▸; Roy *et al.*, 1995 ▸; Kim & Lin, 1996 ▸; Silverman *et al.*, 2004 ▸). Prp2 facilitates the liberation of the 5′ splice site and the branch-site adenosine by destabil­ization of the SF3a and SF3b protein complexes, which are bound to the intron near the branch site. As a consequence, the branch-site adenosine becomes available for subsequent nucleophilic attack of the 5′ splice site (Warkocki *et al.*, 2009 ▸; Lardelli *et al.*, 2010 ▸; Ohrt *et al.*, 2012 ▸; Bao *et al.*, 2017 ▸). Moreover, it has been demonstrated that the remodelling of the spliceosome by Prp2 creates a high-affinity binding site for the NineTeen Complex (NTC) proteins Yju2 and Cwc25, both of which seem to play an important role in stabilizing the pre-mRNA in a suitable conformation for the first transesterification step (Chiu *et al.*, 2009 ▸; Ohrt *et al.*, 2012 ▸; Krishnan *et al.*, 2013 ▸). More recent data also suggest that Prp2 might be directly or indirectly involved in destabilization of the U2/U6 helix Ia and of putative base triples (Wlodaver & Staley, 2014 ▸). The activated spliceosome subsequently undergoes the first catalytic step, generating the C complex.

Recent cryo-EM structures of the B^act^ complex contain Prp2; a homology model derived from the Prp43 crystal structure was used to interpret the cryo-EM map for Prp2 (Rauhut *et al.*, 2016 ▸; Yan *et al.*, 2016 ▸). However, the local resolution of these cryo-EM maps for Prp2 is 7.5 or 9.9 Å; hence, they provide only limited information regarding the Prp2 structure (Ficner *et al.*, 2017 ▸).

Here, we report four crystal structures of Prp2 from *Chaetomium thermo­philum* at resolutions of 1.97, 2.05, 2.3 and 2.7 Å, representing the first high-resolution atomic models of Prp2. One of the crystal structures corresponds to the nucleotide-free state, but it contains a sulfate ion bound in the catalytic centre that probably mimics the remaining phosphate ion after the hydrolysis of ATP and the release of ADP. The other three structures represent Prp2 with a bound ADP molecule, one of which reveals a significantly different ADP conformation.

## 2. Materials and methods   

### 2.1. Gene expression and protein production   

Prp2 from *C. thermophilum* (ctPrp2) was identified by the NCBI *BLAST* search tool (GenBank EGS18341.1) and the gene encoding a truncated version of the protein comprising residues 270–921 was amplified by PCR from a total DNA preparation. The PCR product was cloned into the IBA Stargate pASG-IBA-25 vector according to the manufacturer’s protocol. The GST-fused recombinant protein was expressed in *Escherichia coli* Rosetta 2 (DE3) cells at 16°C. The cells were disrupted using a fluidizer (Microfluidics) in 50 m*M* HEPES–NaOH pH 7.5, 500 m*M* NaCl, 5%(*v*/*v*) glycerol, 10 m*M* EDTA, 1 m*M* DTT and the lysate was clarified by centrifugation for 30 min at 30 000*g*. The protein was purified at 20°C on a Glutathione Sepharose column (GE Healthcare) in 50 m*M* HEPES–NaOH pH 7.5, 500 m*M* NaCl, 5%(*v*/*v*) glycerol, 10 m*M* EDTA, 1 m*M* DTT. A wash step with binding buffer supplemented with 2 *M* LiCl was included to remove bound nucleic acids, followed by elution of the bound fusion protein with 30 m*M* reduced glutathione and cleavage of the GST tag with PreScission protease. Further purification was achieved using a Superdex 200 gel-filtration column, and residual GST was subsequently removed using a Glutathione Sepharose column. A check for contamination by nucleic acids was performed by measuring the absorbance of the protein solution at 260 and 280 nm. The protein was concentrated to 15 mg ml^−1^ using an Amicon Ultra centrifugal concentrator (Merck) in 10 m*M* HEPES–NaOH pH 7.5, 200 m*M* NaCl, 2 m*M* MgCl_2_, 1 m*M* DTT. A shortened construct comprising residues 286–921 was used for the crystallization of crystal form 3 (CF3), but all purification steps were performed as described above.

### 2.2. Crystallization   

Crystallization trials were performed with Prp2 at a concentration of 2 mg ml^−1^ (27.4 µ*M*) and incubated with a tenfold molar excess of ADP or AMPPCP (274 µ*M*). Crystals were grown at 293 K by sitting-drop vapour diffusion using drops consisting of 1 µl protein and 1 µl reservoir solution. Nucleotide-free crystals (NT-free) were obtained in 100 m*M* Tris–HCl pH 7.5, 200 m*M* Li_2_SO_4_, 15%(*w*/*v*) PEG 8000; crystal form 1 (CF1) in 100 m*M* HEPES–MOPS–NaOH pH 7.5, 60 m*M* MgCl_2_/CaCl_2_, 20%(*w*/*v*) ethylene glycol, 10%(*w*/*v*) PEG 8000; CF2 in 100 m*M* Tris–HCl pH 7.5, 200 m*M* NaCl, 15%(*w*/*v*) PEG 8000 and CF3 in 100 m*M* HEPES–NaOH pH 7, 250 m*M* NaCl, 25%(*w*/*v*) PEG 6000. Crystals were obtained after 2–5 d.

### 2.3. Data collection and processing   

The crystals were transferred into mother liquor containing 5%(*v*/*v*) glycerol as well as 10%(*v*/*v*) PEG 400 for CF1 and CF3, and 10%(*v*/*v*) glycerol and 20%(*v*/*v*) PEG 400 for NT-free and CF2 prior to flash-cooling in liquid nitrogen. Oscillation images for NT-free and CF2 were collected on BL14.1 operated by the Helmholtz-Zentrum Berlin (HZB) at the BESSY II electron-storage ring, Berlin-Adlershof, Germany (Mueller *et al.*, 2012 ▸), and the CF1 and CF3 data sets were collected on ID23-2 operated by the European Synchrotron Radiation Facility (ESRF). Data were processed with the *XDS* package using a minimum *I*/σ(*I*) of 1.7 and a minimum CC_1/2_ of 60% for the highest resolution shell as cutting criteria (Kabsch, 2010 ▸).

### 2.4. Structure solution, refinement and analysis   

The crystallographic phase problem was solved by molecular replacement, which was first performed for CF2 with *Phaser* (McCoy *et al.*, 2007 ▸) using chain *A* of scPrp43 (PDB entry 2xau; Walbott *et al.*, 2010 ▸) as a search model. The NT-free, CF1 and CF3 structures were solved using the CF2 structure as a search model for molecular replacement. Manual model building was conducted with *Coot* employing both difference electron-density maps (2*mF*_o_ − *DF*_c_, *mF*_o_ − *DF*_c_) and simulated-annealing composite OMIT maps (Emsley *et al.*, 2010 ▸). Refinements were performed with *PHENIX*, including TLS, weight optimization and bulk-solvent optimization (Adams *et al.*, 2010 ▸). The quality of the final models was assessed using the validation tools in *PHENIX* and *MolProbity* (Chen *et al.*, 2010 ▸). Structure superposition was performed with *LSQMAN* (Kleywegt, 1996 ▸). The figures were prepared with *PyMOL* (v.1.8; Schrödinger). For complete data-collection and refinement statistics, see Table 1 ▸.

### 2.5. Isothermal titration calorimetry (ITC)   

ITC experiments for the binding of ADP and AMPPCP to Prp2 were performed on a MicroCal VP-ITC (Malvern) using a Prp2 concentration in the cell of 10 µ*M* and ADP/AMPPCP concentrations of 100 µ*M* in the syringe. The reaction buffer consisted of 20 m*M* HEPES–NaOH pH 7.5, 200 m*M* NaCl, 5% glycerol, 2 m*M* MgCl_2_. Each measurement consisted of an initial 6 µl injection and 19 injections with a volume of 14 µl injected at a speed of 1 µl s^−1^. The binding was monitored at 20°C and the interval between each injection was set to 250 s. Data integration was performed with *NITPIC*, integrated data were processed and analyzed with *SEDPHAT*, and *GUSSI* was used for the final representation of the data (Houtman *et al.*, 2007 ▸; Keller *et al.*, 2012 ▸; Brautigam, 2015 ▸).

## 3. Results   

### 3.1. Structure of Prp2   

Since all of our attempts to crystallize the spliceosomal DEAH-box ATPase Prp2 from *Saccharomyces cerevisiae* or *Homo sapiens* failed, the orthologue of Prp2 from the ascomycete *C. thermophilum* (ctPrp2) was identified and cloned, as proteins from this thermophilic eukaryotic organism may have a higher tendency to crystallize (Amlacher *et al.*, 2011 ▸). Recent studies of the closely related DEAH-box helicase Prp43 from *C. thermophilum* have proven that proteins from this organism are suitable candidates for structural investigations of spliceo­somal helicases (Tauchert *et al.*, 2016 ▸, 2017 ▸). ctPrp2 exhibits amino-acid sequence identities of 44.1 and 49.2% to yeast Prp2 (scPrp2) and human Prp2 (hsPrp2), respectively. However, full-length ctPrp2 was not soluble after overproduction in *E. coli*; hence, an N-terminally truncated version of ctPrp2 comprising residues 270–921 was cloned, expressed, purified and crystallized. Notably, the missing 269 N-terminal residues were known to not be essential for Prp2 function in yeast (Edwalds-Gilbert *et al.*, 2004 ▸). Four different crystal structures of ctPrp2 were obtained: one of the nucleotide-free state and three different crystal forms of the ADP-bound state (Table 1 ▸). The nucleotide-free structure was the result of crystallization trials in the presence of the nonhydrolysable ATP analogue AMPPCP and is referred to here as NT-free, whereas the nucleotide-bound structures are denoted crystal forms 1–3 (CF1–CF3).

**Figure 1:**
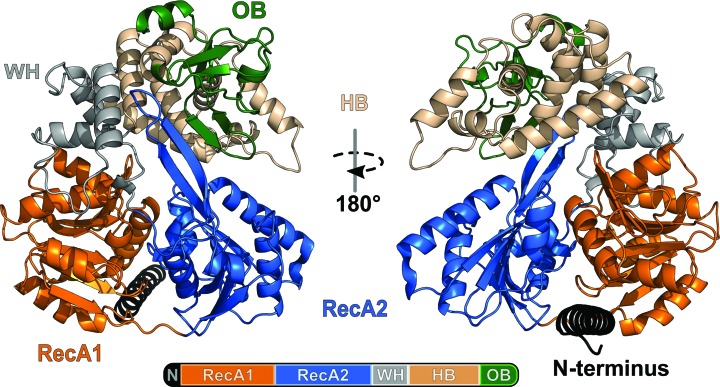
Overall structure of Prp2 from *C. thermophilum*. The model of ctPrp2 is depicted as a cartoon. The remaining amino acids from the truncated N-terminal extension (270–296) are shown in black, the RecA1 domain (297–475) is in orange, the RecA2 domain (476–652) is in blue, the winged-helix domain (WH; 653–720) is in grey, the helix-bundle domain (HB; 721–852) is in wheat and the oligonucleotide-binding fold (OB; 853–920) is in green.

Full-length Prp2 consists of six domains, five of which are present in the ctPrp2 structure (Fig. 1 ▸ *a*) as the 269-residue N-terminal domain is absent in the truncated protein used for crystallization. The helicase core comprises two RecA-like domains (referred to as RecA1 and RecA2; Fig. 1 ▸ *a*). RecA1 contains the conserved Walker motif A (P-loop) between residues 320 and 327. RecA2 contains a prominent antiparallel β-hairpin that protrudes out of the RecA2 domain. The winged-helix (WH) and helix-bundle (HB) domains are located C-terminally to RecA2; both are homologous to the corresponding domains in Prp43 and the Ski2-like DNA helicase Hel308 (Büttner *et al.*, 2007 ▸; Richards *et al.*, 2008 ▸). The helix-bundle domain was previously denoted as a ratchet domain and was named according to its previously proposed function of acting as a ratchet in dsDNA or dsRNA unwinding (Walbott *et al.*, 2010 ▸; He *et al.*, 2010 ▸); this however turned out not to be the case, as shown for other RHA and DEAH-box proteins (Prabu *et al.*, 2015 ▸; Tauchert *et al.*, 2017 ▸). The C-terminal domain includes a five-stranded β-barrel exhibit­ing an oligonucleotide-binding fold (OB-fold; Murzin, 1993 ▸). These five domains are common to all spliceosomal DEAH-box helicases.

**Figure 2:**
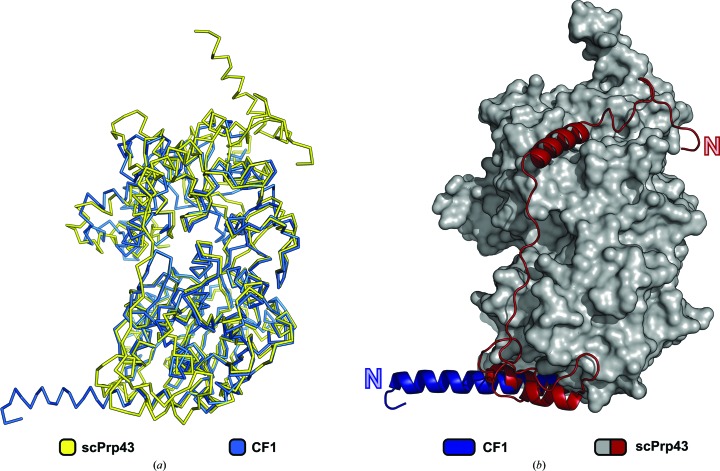
Structural comparison of ctPrp2 and scPrp43 (PDB entry 3kx2; He *et al.*, 2010 ▸). (*a*) Superposition of ctPrp2 (blue) and scPrp43 (yellow), depicted here as ribbon models, reveals a high structural similarity, resulting in an r.m.s.d. of 1.3 Å for 573 common C^α^ atoms. (*b*) The most prominent difference is found at the N-termini of the proteins. The N-termini of ctPrp2 and scPrp43 are shown as cartoons, whereas the protein core of scPrp43 is shown as a surface (grey). The N-terminal extension of ctPrp2 (blue) protrudes from the protein core, whereas the N-terminus of scPrp43 (red) wraps around the RecA1 domain and parts of the C-terminal domains.

In comparison to the known structure of the DEAH-box helicase Prp43, the fold of Prp2 is very similar, with the exception of the N-terminal extension (Fig. 2 ▸). In scPrp43 the N-terminal extension wraps around the RecA1 domain and the N-terminal residues bind to the C-terminal domains (Walbott *et al.*, 2010 ▸; He *et al.*, 2010 ▸). In contrast, in Prp2 the 26 residues N-terminal to the RecA1 domain form a long α-helix that protrudes from the globular protein molecule. The first three turns of this helix superimpose well with the corresponding helix in Prp43. However, in Prp43 this helix terminates after 13 residues because of a helix-breaking proline at position 81.

### 3.2. Structural flexibility of the C-terminal domains   

**Figure 3:**
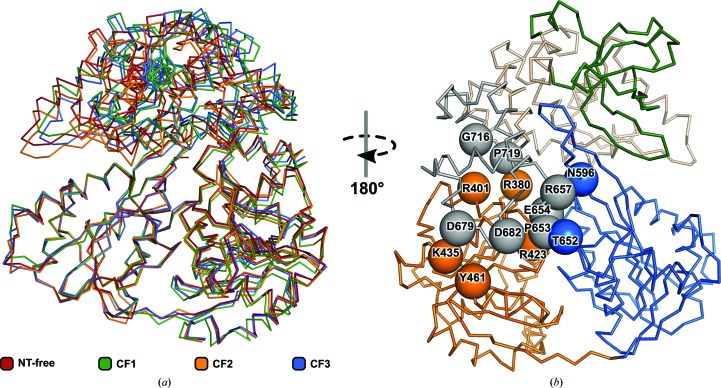
Flexibility of the C-terminal entity. (*a*) A superposition of all of the ctPrp2 structures *via* the RecA2 domain highlights the flexibility of the C-terminal domains with respect to the RecA2 domain. While the helicase cores superpose well, the C-terminal domains reveal various positions. (*b*) Despite the observed flexibility, ten common interdomain interactions between the C-terminal domains and the helicase core can be found among the four structures. The residues involved in these conserved interactions are displayed as spheres and labelled accordingly. ctPrp2 is shown as a ribbon model and the domains are coloured according to Fig. 1 ▸.

Superpositions of the four Prp2 crystal structures presented here reveal distinguishable differences between their conformations, resulting in overall root-mean-square deviations (r.m.s.d.s) calculated between backbone C^α^ atoms (residues 286–920) that range between 0.81 and 1.70 Å (the average r.m.s.d. based on six superpositions is 1.37 Å). This is significantly larger than the backbone r.m.s.d.s calculated separately for the RecA2 domain (residues 471–595 and 611–652), the helicase core comprising both RecA-like domains (residues 286–595 and 611–652), and the C-terminal domains (WH, HB and OB; residues 653–920), which range from 0.50 to 0.86 Å (average 0.65 Å), from 0.66 to 1.24 Å (average 0.97 Å) and from 0.44 to 1.03 Å (average 0.86 Å), respectively. Thus, keeping the RecA2 domains of the four compared Prp2 structures superimposed, the observed differences in the positions of their C-terminal domains (CTDs; WH, HB and OB) relative to the helicase core become more clear (Fig. 3 ▸ *a*).

The observed movement of the C-terminal domains (CTDs) with respect to the helicase core results in a different number of interdomain interactions (Supplementary Table S1). Interestingly, ten of these interactions, involving residues from the winged-helix domain (Supplementary Table S1, Fig. 3 ▸ *b*), are conserved among the four analyzed Prp2 structures regardless of the movement of the CTDs. The residues taking part in these conserved interactions are Arg657, Pro653 and Glu654, which form hydrogen bonds to Asn596 and Thr652 from the RecA2 domain; Pro719, Gly716, Glu654 and Asp679, which form polar contacts with Arg380, Arg401, Arg423 and Tyr461 from the RecA1 domain; and Arg423 and Lys435, which form salt bridges with Asp682 and Asp679 located in the RecA2 domain, respectively. This network of interactions between the winged-helix (WH) domain and the helicase core anchors the CTDs to the adjacent surface of the RecA1 domain. As a consequence, any movement of the RecA1 domain relative to the RecA2 domain triggers changes in the position of the CTDs, which form a single entity with the RecA1 domain. Surprisingly, comparison of the four Prp2 structures revealed that the observed changes in the position of the CTDs are not related to the presence or absence of ADP (Fig. 3 ▸ *a*).

### 3.3. Variable β-hairpin conformation   

**Figure 4:**
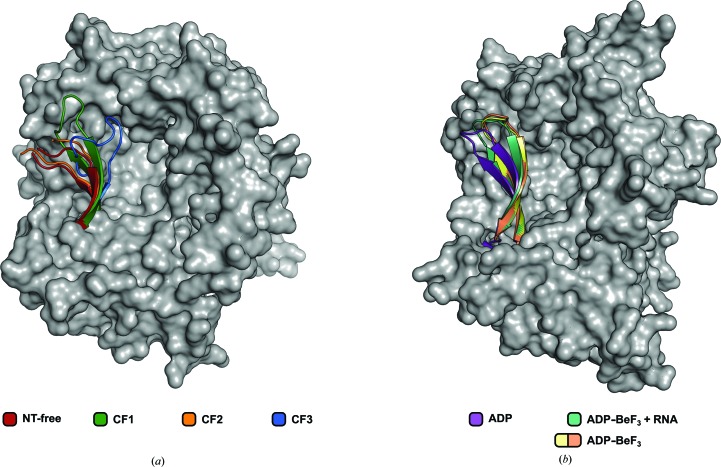
Comparison of the different β-hairpin conformations. (*a*) All ctPrp2 structures are superposed *via* their RecA2 domains. The different β-hairpins are presented as cartoon models using the colouring of Fig. 3 ▸(*a*), while the remaining part of the protein is shown as a surface (nucleotide-free structure only). The OB-fold domain was omitted for clarity. The superposition reveals that the β-hairpin of ctPrp2 can adopt different conformations. (*b*) Superposition of ctPrp43 structures in different functional states *via* their RecA2 domains (PDB entries 5d0u, 5lta, 5ltj and 5ltk; Tauchert *et al.*, 2016 ▸, 2017 ▸). The structures are presented according to (*a*) using different colours.

A superposition of the four compared Prp2 structures based on their RecA2 domains unveiled structural flexibility of the C-terminal domains and their coupled movement with the RecA1 domain. A closer look at the four superimposed RecA2 domains (Fig. 4 ▸ *a*) shows substantial differences in the conformation of the β-hairpin (residues 596–610), resulting in a significant increase in the calculated r.m.s.d.s, which range from 0.71 to 2.15 Å (average 1.61 Å) when compared with the r.m.s.d.s calculated between RecA2 domains without this flexible fragment (0.50–0.86 Å, average 0.65 Å). The observed conformational flexibility of the β-hairpin leads to substantial differences in the number of interdomain interactions formed between this structural element and mainly the winged-helix and OB-fold domains (Supplementary Table S1). The β-hairpins of NT-free and CF2 have similar conformations and protrude out of the molecular scaffold (Fig. 4 ▸ *a*). The tips of these two β-hairpins were not resolved in the electron-density map and are probably disordered. In contrast, the β-hairpins of CF1 and CF3 are fully resolved and exhibit significantly different conformations and numbers of interdomain contacts (Supplementary Table S1). The largest number of interactions was observed for the deeply buried β-hairpin of CF3, which interacts mostly with the C-terminal domains.

A thorough analysis of the structural superposition of the four Prp2 structures reveals an apparent correlation between the conformation of the β-hairpin and the position of the C-terminal domains with respect to the helicase core (Fig. 4 ▸ *a*). Prp2 structures with the β-hairpin protruding out of the protein scaffold (NT-free and CF2) exhibit CTDs closer to the surface of the helicase core. Conversely, the structure with the buried β-hairpin (CF3) exhibits the most distant position of the CTDs relative to the RecA domains.

A comparable correlation can be observed in the structures of *C. thermophilum* Prp43. These structures exhibit significant conformational changes of the C-terminal domains which are related to their individual functional states (Tauchert *et al.*, 2016 ▸, 2017 ▸). It has been shown that the C-terminal domains are positioned closest to the helicase core in the ADP-bound state, are slightly more distant in the RNA/ADP-BeF_3_-bound state and are by far the furthest apart in the ADP-BeF_3_-bound state. Although the degree of movement of the β-hairpin in these structures is decreased and the conformational divergence of the C-terminal domains is much more pronounced when compared with the structures of Prp2 presented here, it is notable that the most distal β-hairpin is present in the ADP-bound state, while the most integrated β-hairpins are found in the ADP-BeF_3_-bound state (Fig. 4 ▸ *b*).

### 3.4. Alternate ADP conformations   

The two RecA-like domains form a cleft harbouring the bound ADP nucleotide. In the reported crystal structures of ctPrp2 complexed with ADP, the nucleoside diphosphates are bound by Gly323, Gly325, Lys326, Thr327 and Thr328. An Mg^2+^ ion is coordinated by four water molecules, a β-phosphate O atom and the hydroxyl group of the Thr327 side chain.

**Figure 5:**
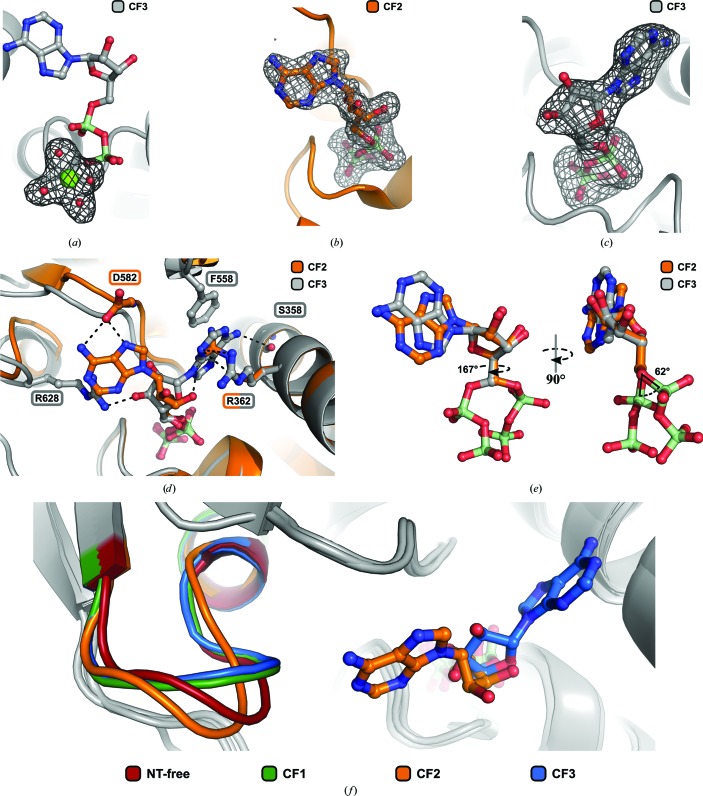
Comparison of differently bound ADP molecules. ADP molecules are presented as ball-and-stick models and the magnesium ion and water molecules as spheres. ctPrp2 is depicted as a cartoon representation with highlighted residues as ball-and-stick models. (*a*) depicts the *mF*_o_ − *DF*_c_ electron-density OMIT map for the magnesium ion with the four coordinated waters of CF3 (grey) contoured at 3σ. This OMIT map is representative of all ADP-bound structures. The OMIT maps at the same contour level for the ADP molecules of CF2 (orange) and CF3 are displayed in (*b*) and (*c*), respectively. (*d*) On superposing the RecA1 domains of CF2 and CF3 the phosphate moieties align almost identically, but the adenosine is present in a previously undescribed conformation, leading to different adenosine interactions in CF2. (*e*) A superposition of the different ADP molecules *via* the ribose reveals that the adenine is present in a *syn* (CF2) and an *anti* (CF3) conformation and that the torsions along the C4′—C5′ bond and the C5′—O5′ bond are changed by 167 and 62°, respectively. (*f*) Depending on the ADP conformations described in (*d*) and (*e*), a loop in the nearby motif VI adopts distinct conformations. A superposition of the RecA2 domains reveals a flipped conformation of this loop in the NT-free and CF2 structures compared with CF1 and CF3, which exhibit an ADP-binding pattern comparable to other DE*x*D/H-box helicases.

Despite this similarity, the conformation of the adenosine and its binding mode differ significantly between CF1/CF3 and CF2 (Fig. 5 ▸ *c*). In CF1 and CF3 the adenine is sandwiched between Arg362 and Phe558 and the N6 atom forms a hydrogen bond to Ser358. The 3′-OH group of the ribose is involved in a polar interaction with Arg628. This conformation of the bound adenosine in the *anti* conformation in CF1/CF3 is very similar to that observed in Prp43–ADP crystal structures (Tauchert *et al.*, 2016 ▸; Walbott *et al.*, 2010 ▸; He *et al.*, 2010 ▸).

In CF2, a novel conformation of the adenosine has been observed which is unique among all known DE*x*D/H-box helicase structures. While the conformation and binding mode of the diphosphate moiety remained unchanged, the base is flipped over into the *syn* conformation and points towards the RecA2 domain (Fig. 5 ▸ *d*). In this new conformation (CF2) the adenine moiety forms two polar interactions with Asp582. The side chain of Arg362 is repositioned, now occupying the position of the adenine in CF1/CF3, forms a hydrogen bond to the 3′-OH group of the ribose. Superposition of the two ADP molecules (CF1 and CF2) based on their ribose moieties clearly depicts the actual differences in conformations (Fig. 5 ▸ *e*). Although the sugar pucker remained unchanged (C2′-*endo* conformation), the torsion angles of the C4′—C5′ and C5′—O5′ bonds are changed in the CF2 conformation relative to those in CF1/CF3 by 167 and 62°, respectively. The observed conformational heterogeneity of ADP in the reported Prp2 crystal structures (CF2 *versus* CF1/CF3) reveals an intrinsic flexibility of the helicase core which is not related to large structural rearrangements or to crystal packing (CF3 and CF2 are isostructural).

Additionally, the novel binding mode of ADP is accompanied by a change in the conformation of a loop at the C-terminal end of the conserved sequence motif VI (Fig. 5 ▸ *d*). A superposition of the RecA2 domains reveals that these loops in CF1 and CF3 adopt almost identical conformations and point towards ADP, whereas in CF2 this loop flips into a position more distant from the ADP. This alternate conformation of the loop enables the repositioning of the adenine as seen in CF2, as otherwise Arg628, which interacts with the ribose in CF1 and CF3, would hinder the novel conformation of ADP (Fig. 5 ▸ *c*). Interestingly, this loop occupies a middle position in the nucleotide-free Prp2 structure when compared with the CF2 and CF1/CF3 Prp2–ADP structures.

### 3.5. Nucleotide-free Prp2   

**Figure 6:**
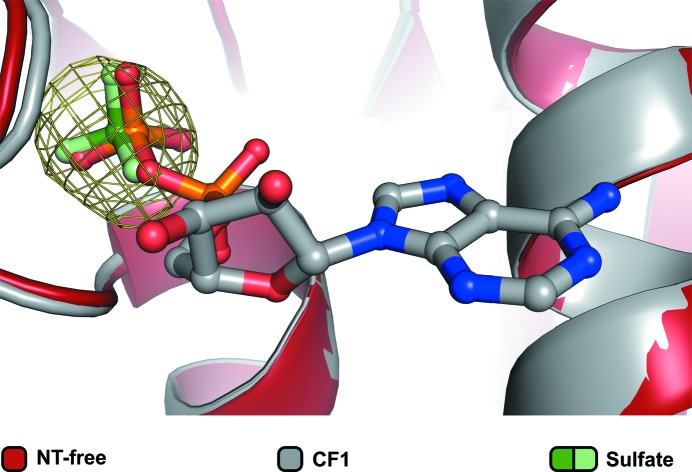
The nucleotide-free Prp2 structure with a bound sulfate molecule. A superposition of the RecA1 domains of the NT-free and CF1 structures shows that a sulfate that is present at the active site of the nucleotide-free structure adopts the same position as the β-phosphate of the bound ADP.

The nucleotide-free Prp2 contains a sulfate ion that occupies the position of the β-phosphate in the ADP-complex structures (Fig. 6 ▸). The O atoms of the sulfate ion form a similar network of interactions as the β-phosphates of the ADP-bound structures, namely with Gly323, Gly325, Lys326 and Thr327. The overall structure of nucleotide-free Prp2 more closely resembles that of CF2: the C-terminal domain is positioned close to the helicase core and the partially dis­ordered β-hairpin protrudes out of the protein core (Fig. 3 ▸ *a*). In contrast to CF1 and CF3, the motif VI loop is slightly flipped away from the ADP-binding pocket (Fig. 5 ▸ *d*).

The nucleotide-free structure was the result of crystallization trials of Prp2 with the nonhydrolysable ATP analogue AMPPCP. In order to explain the lack of bound AMPPCP, binding studies of AMPPCP and ADP were conducted using isothermal titration calorimetry (ITC). This experiment resulted in no significant heat signal with the buffer conditions used when AMPPCP was titrated. Although the lack of thermal signal might also arise from enthalpy compensation in the case of a deprotonation upon binding, this result strongly suggests that Prp2 does not bind AMPPCP (Supplementary Fig. S1*a*). This finding is in agreement with the absence of AMPPCP in the nucleotide-free structure. The terminal O atom of the ADP in CF1–CF3 is involved in a hydrogen bond to Gly323 and this interaction could not take place with the methylene bridge of AMPPCP, which might explain the lack of binding of this ATP analogue. Binding studies *via* ITC were also performed for ADP as a control, and a dissociation constant (*K*_d_) of 179 n*M* was obtained (Supplementary Fig. S1*b*).

## 4. Discussion   

Here, we report the first crystal structures of Prp2 in two different functional states (nucleotide-free and ADP-bound). The atomic models of both functional states share a very similar conformation and their overall structures strongly resemble the structure of the closely related DEAH-box helicase Prp43 (Fig. 2 ▸). However, despite these structural similarities, Prp2 and Prp43 differ significantly in their function. In contrast to all other spliceosomal DEAH-box proteins, no helicase activity has been observed for Prp2 *in vitro* (Warkocki *et al.*, 2015 ▸; Bao *et al.*, 2017 ▸). This atypical lack of helicase activity appears to be consistent with the function of Prp2 as deduced from the recently determined cryo-EM structures of the B^act^ complex (Rauhut *et al.*, 2016 ▸; Yan *et al.*, 2016 ▸). Prp2 is located on the periphery of the B^act^ spliceosome, contacting the outer side of the Hsh155 HEAT repeats, and therefore Prp2 is rather distant from the 5′ splice site and the branch-point adenosine (Supplementary Fig. S4). This is in agreement with previous biochemical data showing that Prp2 binds to the single-stranded intron ∼30 nt downstream of the branch-point site (Liu & Cheng, 2012 ▸; Warkocki *et al.*, 2015 ▸). All data to date suggest that Prp2 does not act as a helicase by unwinding double-stranded RNAs, but rather functions as an RNA-dependent RNPase. The analysis of different functional states of Prp2 might provide insight into the peculiar manner of function of this spliceosomal DEAH-box helicase.

Structures of Prp2 in the nucleotide-free state and in the ADP-bound state are presented in this study. The latter is likely to represent the state of the DEAH-box protein after ATP hydrolysis and can thus be considered as a post-catalytic structural snapshot of this protein. Two of the three ADP-bound Prp2 structures (CF1/CF3) not only show a high overall structural similarity to the known post-catalytic state of the DEAH-box helicase Prp43, but also exhibit a comparable ADP conformation (Fig. 2 ▸ *a*). Interestingly, the third ADP-bound structure (CF2) reveals a previously undescribed ADP conformation. In this structure the phosphates are bound in the same manner as in CF1/CF3, but the adenosine is flipped away from its position sandwiched between the RecA domains and points towards the RecA2 domain (Fig. 5 ▸ *d*). This unique conformation is a consequence of changes in the torsion angles of the C4′—C5′ and C5′—O5′ bonds of 167 and 62°, respectively (Fig. 5 ▸ *e*). Additionally, the adenine exhibits a *syn* conformation in CF2, in contrast to the more common *anti* conformation that is present in CF1 and CF3. The conformational changes of the ADP in CF2 seem to be accompanied by a flip in a neighbouring loop at the C-terminal end of motif VI and can only be sustained upon its conformational change, as otherwise the adenine would clash with Arg628 (Figs. 5 ▸ *d* and 5 ▸ *f*). Additionally, the more distal position of the loop slightly enlarges the entry to the nucleotide-binding pocket, which has also been observed in the nucleotide-free structure. The widening of the entry site to the binding cleft could play a role in the initial binding of ATP or the release of ADP, as it might make the binding pocket more easily accessible to the ATP molecule or promote the expulsion of ADP after completed hydrolysis. A further indication that the observed atypical ADP conformation might correspond to the ADP-prerelease state can be based on an analysis of the interactions between Prp2 and the adenosine moiety. Compared with the typical conformation, two hydrogen bonds (Ser358–N6 and Arg628–3′-OH), a π–π interaction and a cation–π interaction are exchanged for three polar interactions (Asp582–N6/N7 and Arg362–3′-OH; Fig. 5 ▸ *d*). The reduced number of interactions formed by the adenosine suggests a more loose association of the nucleotide with the helicase, which thereby also abandons its position in the interface between the two RecA domains. Thus, the Prp2 structure with the flipped ADP might correspond to a structural snapshot subsequent to the state observed in the other two ADP-bound states and prior to ADP release. The nucleotide-free structure could represent the state of Prp2 immediately after the release of ADP as well as shortly before the uptake of ATP. CF1 and CF3, on the other hand, display an ADP conformation that is already known from Prp43 structures, in which the adenine is sandwiched between an arginine from the RecA1 domain and a phenylalanine from the RecA2 domain (He *et al.*, 2010 ▸; Walbott *et al.*, 2010 ▸; Tauchert *et al.*, 2016 ▸). These residues have been shown to be crucial for the NTPase and helicase activity of Prp43, in which the phenyl­alanine seems to play a role in coupling ATPase activity to helicase activity and an arginine-to-alanine mutant impairs both functions (Robert-Paganin *et al.*, 2017 ▸). The substantial role of these two residues in the catalytic processes of Prp43 suggests that the ADP-bound structures with the adenine sandwiched between these two amino acids might represent the state directly subsequent to catalysis.

Interestingly, no large structural rearrangements were observed between the structures of the nucleotide-free and ADP-bound states of Prp2, which share an almost unchanged nucleotide-binding pocket. The bound sulfate ion seems to mimic the β-phosphate of ADP (Fig. 6 ▸), as both groups share approximately the same position and an identical network of interactions. On the other hand, ADP and P_i_ are generated after ATP hydrolysis, and to date no exact order of events after this step has been assigned for DEAH-box helicases. For the DEAD-box helicase Dbp5 it was demonstrated that release of P_i_ is the rate-limiting step and is then followed by ADP release (Wong *et al.*, 2016 ▸). Despite the high similarity of the helicase cores of these two families, DEAD-box helicases lack the C-terminal domains of the DEAH-box family, and the RecA domains can adopt so-called open and closed conformations, with the former resulting in a disrupted ATP-binding pocket (Ozgur *et al.*, 2015 ▸). In contrast, the DEAH-box helicases exhibit restricted movement of the RecA domains which results in the adoption of a permanent quasi-closed state with a preformed nucleotide-binding pocket. This difference in the mode of action might lead to different catalytic mechanisms and makes it difficult to predict whether helicases belonging to the DEAH-box and DEAD-box families act in the same manner. Since the RecA domains always stay in close proximity to each other in DEAH-box helicases, the nucleotide-binding site is completely blocked by the bound ADP molecule after catalysis. Thus, if P_i_ is released before ADP a separate exit passage is necessary. Such an exit passage has not so far been identified in structures of this helicase family, and this makes it an interesting target for future studies. Since the nucleotide-free structure is likely to represent Prp2 after ADP release, the bound sulfate ion might mimic the remaining phosphate ion, which can now be released *via* the same passage as used for ADP exit/ATP entry. Although the reported structures might provide a hint to the sequence of events upon ATP hydrolysis, detailed biochemical analyses, as have been performed for the DEAD-box family, are required in order to completely understand the catalytic mechanism of DEAH-box proteins.

Recently, several crystal structures of Prp43 in the pre­catalytic state have been reported and have revealed how ATP analogues and RNA bind to DEAH-box helicases; they have also allowed the proposal of a sequence of events prior to ATP hydrolysis (Tauchert *et al.*, 2017 ▸; He *et al.*, 2017 ▸). In the proposed model, binding of ATP triggers a dramatic movement of the C-terminal domains relative to the RecA domains, leading to opening of the RNA-binding tunnel, which was previously completely enclosed. Once the C-terminal domains are in a more distant position with regard to the helicase core, the RNA is then able to be incorporated into the RNA-binding tunnel, which closes again upon binding RNA, circumventing the need for a threading-like integration of the RNA. The varying number of contacts, especially between the HB/OB and the helicase core, highlights the flexibility of the C-terminal domains and is in accordance with this mechanism (Supplementary Table S1; Fig. 3 ▸ *a*). However, on the opposite side of the RNA entrance the C-terminal domains need to be stably connected to the helicase core, and in Prp2 ten interdomain interactions were found that are present throughout all four structures (Fig. 3 ▸ *b*, Supplementary Table S1). The WH domain is the only C-terminal domain that is involved in these conserved interactions and thus represents the most stable anchor point. Considering the facts that these common interactions are also conserved in Prp43 and that the observed flexibility of the C-terminal domains would support the current model of RNA binding, it seems likely that this mechanism might also apply to Prp2. Additionally, analysis of the Prp2 structures revealed a correlation between the position of the β-hairpin and the location of the C-terminal domains. The structures with a protruding β-hairpin exhibit a conformation of the C-terminal domains that is closer to the surface of the helicase core (Figs. 3 ▸ *a* and 4 ▸ *a*). This correlation suggests a passive role of the β-hairpin to be unlikely as it would otherwise be coupled to the movement of the C-terminal domains. Interestingly, a similar correlation can be found in the *C. thermophilum* Prp43 structures, although the conformational differences of the β-hairpins are smaller and the movement of the C-terminal domains is more pronounced (Fig. 4 ▸ *b*). Based on structural comparisons with the Ski2-like DNA helicase Hel308 it has been proposed that this β-hairpin might play a role in duplex melting, but since no detailed unwinding mechanism for DEAH-box members has been described to date, the exact purpose of this structural feature remains unclear (Büttner *et al.*, 2007 ▸). The presented structures of Prp2 might link the repositioning of the β-hairpin to the movement of the C-terminal domains, but either structures of DEAH-box proteins with dsRNA or in-depth biochemical studies are needed to shed light onto the function of the β-hairpin.

The N-terminal extension of Prp2 has been shown not to be essential for its function, and the constructs used almost completely lacked this domain (Edwalds-Gilbert *et al.*, 2004 ▸). In contrast to the high sequence and structural similarity of the helicase core and C-terminal domains among DEAH-box proteins, the conservation of the N-terminal extensions is significantly lower and they have also been proposed to play a role in various noncatalytic functions such as regulation and recruitment (Supplementary Fig. S3; Tauchert *et al.*, 2016 ▸; Wang & Guthrie, 1998 ▸; Schneider & Schwer, 2001 ▸). Although the major part of the N-terminal extension of Prp2 (269 of 296 amino acids) is missing in the constructs used for crystallization, a clear difference from the only structurally known genuine DEAH-box helicase Prp43 can already be found. The N-terminal extension of Prp43 consists of three short α-helices and wraps around the globular protein, whereas the part of the N-terminal domain that is present in the Prp2 crystal structures shows a long α-helix that protrudes out of the protein core (Fig. 2 ▸ *b*). Although it is involved in crystal contacts with two symmetry-related molecules, secondary-structure predictions from numerous servers agree on the presence of an α-helix between residues 270 and 296, as can be seen in CF1 (Supplementary Fig. S5; Kurowski & Bujnicki, 2003 ▸). This protruding helix represents the last residues of the N-terminal domain, which reaches out in order to make contacts with other spliceosomal factors distant from the main body of the helicase. This scenario is in agreement with cryo-EM data and cross-linking studies, revealing putative interaction sites of this N-terminal extension with Prp45 (Prp2 Lys101/102/120/128 and Prp45 Lys274; Rauhut *et al.*, 2016 ▸; Yan *et al.*, 2016 ▸). However, owing to the high flexibility of these peripheral B^act^ spliceosome regions no detailed structural information for these interaction sites could be obtained to date. The distance between the beginning of the Prp2 N-terminal α-helix and residue Ala292, which is positioned in the closest vicinity to the cross-linking site of Prp45, amounts to roughly 110 Å. This spatial separation can be bridged by an N-terminal extension of at least 220 amino acids (220 in *S. cerevisiae*, 296 in *C. thermophilum* and 390 in *H. sapiens*), which is predicted to have a high α-helical content. The last 26 residues of this domain, which are present in CF1, also adopt an α-helical conformation. Interestingly, in human Prp2 (DHX16) the most N-terminal tip of the corresponding domain has been proposed to contain a PWI-like domain, which is also present in the spliceosomal helicases Brr2 (U5-200K) and has been predicted for Prp22 (DHX8) (Absmeier *et al.*, 2015 ▸; Korneta *et al.*, 2012 ▸). In Brr2 the PWI-like domain-containing N-terminal region has been proposed to act as a structural switch autoinhibiting Brr2 *via* substrate competition and conformational clamping (Absmeier *et al.*, 2015 ▸; Agafonov *et al.*, 2016 ▸; Nguyen *et al.*, 2016 ▸). In the absence of detailed structural and biochemical information on the N-terminal extension of Prp2, one can only speculate about the functional role of this domain. The presumably distant location from the catalytically active protein core seems to exclude it from catalysis-related functions, with it rather being responsible for recruitment and proper positioning in the spliceosome.

Despite the many parallels between Prp2 and Prp43 discussed in this structural study, the key question of why Prp2 is not able to act as an actual helicase cannot be answered using the presented structures. Prp2 and Prp43 are known to interact with so-called G-patch proteins and the regulation of their functions is tightly linked to this interaction. Detailed biochemical as well as structural characterization of this interaction might help to understand the peculiar manner of function of Prp2. However, most importantly, structural insights into Prp2 in a precatalytic state with bound RNA and an ATP analogue might reveal differences from the existing precatalytic structures of Prp43 which could shed light onto the as yet unknown mechanism of action of Prp2.

## 5. Related literature   

The following references are cited in the Supporting Information for this article: Fica *et al.* (2017 ▸) and Liu *et al.* (2017 ▸). 

## Supplementary Material

PDB reference: DEAH-box helicase Prp2, 6fa9

PDB reference: complex with ADP, crystal form 1, 6fac

PDB reference: crystal form 2, 6faa

PDB reference: crystal form 3, 6fa5

Supplementary Figures and Table.. DOI: 10.1107/S2059798318006356/ud5003sup1.pdf

## Figures and Tables

**Table 1:** Data-collection and refinement statistics

	NT-free	CF1	CF2	CF3
Data collection				
Space group	*P*2_1_2_1_2_1_	*C*2	*P*2_1_2_1_2_1_	*P*2_1_2_1_2_1_
Unit-cell parameters				
*a* (Å)	50.6	122.7	50.3	48.2
*b* (Å)	114.1	67.3	114.2	116.4
*c* (Å)	120.7	95.6	117.8	118.2
β (°)	90	106.5	90	90
X-ray source	BL14.1, BESSY	ID23-2, ESRF Grenoble	BL14.1, BESSY	ID23-2, ESRF Grenoble
Resolution range (Å)	41.46–2.60 (2.70–2.60)	46.41–2.05 (2.15–2.05)	46.00–1.97 (2.07–1.97)	44.64–2.30 (2.44–2.30)
No. of unique reflections	22177	46873	48358	30007
Completeness (%)	99.8 (99.9)	99.5 (99.6)	99.0 (99.3)	99.1 (97.9)
*R*_merge_ (%)	7.0 (84.0)	3.9 (79.4)	9.2 (84.6)	7.9 (85.3)
Average *I*/σ(*I*)	20.43 (2.34)	20.31 (2.19)	13.43 (1.77)	13.78 (1.91)
Multiplicity	4.86 (4.98)	4.08 (4.2)	3.74 (3.6)	4.28 (4.35)
CC_1/2_ (%)	99.9 (82.5)	99.9 (83.4)	99.7 (62.7)	99.8 (65.3)
Wilson *B* (Å^2^)	50.97	44.79	24.74	49.4
Refinement				
Resolution (Å)	41.46–2.70 (2.85–2.70)	46.41–2.05 (2.09–2.05)	46.00–1.97 (2.01–1.97)	44.64–2.30 (2.38–2.30)
No. of reflections	22168	46832	48349	29999
*R*_work_ (%)	23.00 (32.89)	21.60 (35.53)	18.75 (29.83)	19.79 (31.56)
*R*_free_ (%)	27.59 (33.99)	24.41 (37.63)	22.42 (32.82)	24.54 (35.45)
Total No. of atoms	5020	5384	5553	5047
Protein residues	636	653	637	635
Water molecules	42	222	445	59
Ligand molecules	2	6	7	3
R.m.s. deviations				
Bond lengths (Å)	0.004	0.010	0.005	0.006
Bond angles (°)	0.852	1.476	1.051	0.792
Mean *B* factors (Å^2^)				
Protein	70.2	62.7	29.3	58.2
ADP	—	58.6	25.7	57.4
Ramachandran statistics				
Favoured (%)	97.15	97.55	98.13	97.00
Allowed (%)	2.69	2.45	1.72	3.00
Outliers (%)	0.16	0.0	0.16	0.00
PDB code	6fa9	6fac	6faa	6fa5
