# Supplementary material for: Crystal structure of the spliceosomal DEAH-box ATPase Prp2
Source: Acta Crystallogr D Struct Biol. 2018 Jun 8;74(Pt 7):643–54. doi: 10.1107/S2059798318006356 (PMC6038383; doi:10.1107/S2059798318006356)
Supplement: Supplementary file 1 [file d-74-00643-sup1.pdf]

STRUCTURAL BIOLOGY

Volume 74 (2018)

Supporting information for article:

Crystal structure of the spliceosomal DEAH-box ATPase Prp2

Andreas Schmitt, Florian Hamann, Piotr Neumann and Ralf Ficner

Table S1 Overview of interdomain interactions. Contacts between the helicase core and the C-terminal domains are listed for each structure and residues are colorcoded according to Fig. 1. Salt bridges (SB) are highlighted with a cross.

Interactions	Apo HC	Apo CTD	Apo SB	CF1 HC	CF1 CTD	CF1 SB	CF2 HC	CF2 CTD	CF2 SB	CF3 HC	CF3 CTD	CF3 SB
Conserved Interdomain Interactions	Arg380	Pro719		Arg380	Pro719		Arg380	Pro719		Arg380	Pro719	
	Arg401	Gly716		Arg401	Gly716		Arg401	Gly716		Arg401	Gly716	
	Arg423	Glu654		Arg423	Glu654		Arg423	Glu654		Arg423	Glu654	
	Arg423	Asp682	X	Arg423	Asp682	X	Arg423	Asp682	X	Arg423	Asp682	X
	Lys435	Asp679	X	Lys435	Asp679	X	Lys435	Asp679	X	Lys435	Asp679	X
	Tyr461	Asp679		Tyr461	Asp679		Tyr461	Asp679		Tyr461	Asp679	
	Asn596	Arg657		Asn596	Arg657		Asn596	Arg657		Asn596	Arg657	
	Thr652	Pro653		Thr652	Pro653		Thr652	Pro653		Thr652	Pro653	
	Thr652	Arg657		Thr652	Arg657		Thr652	Arg657		Thr652	Arg657	
	Thr652	Glu654		Thr652	Glu654		Thr652	Glu654		Thr652	Glu654	
β-hairpin Interactions	Glu595	Arg657	X	Glu595	Arg657	X	Gly605	Asn870		Glu595	Arg657	X
	Thr597	Arg657		Phe598	Arg657		Gly605	Lys691		Asn596	Glu654	
	Gln606	Asn870		Val601	Asn870		Gln606	Lys869		Asn596	Thr658	
	Ser607	Arg657		Gly602	Lys691		Gln606	Asn870		Thr597	Arg657	
	Ser607	Ser659		Thr603	Asn694		Ser607	Arg657		Phe598	Arg657	
	Leu609	Leu897		Thr603	Lys869		Thr608	Leu897		Pro600	Lys691	
	Leu609	Val899		Gln606	Asn870		Leu609	Leu897		Pro600	Asn870	
	Val611	Val899		Leu609	Leu897		Leu609	Val899		Val601	Tyr894	
				Leu609	Val899		Val611	Val899		Thr603	Asn854	
										Thr603	His895	
										Thr604	Ser659	
										Thr604	Ser661	
										Gly605	Ser662	
										Gln606	Glu721	
										Gln606	His895	
										Val611	Val899	
Remaining Interactions	Phe381	Gln818		Arg401	Gln666		Arg353	Arg811		Phe381	Gln818	
	Arg401	Gln666		Arg401	Glu717		Glu382	Arg811	X	Val425	Glu654	
	Val425	Glu654		Arg401	Pro719		Arg401	Gln666		Lys457	Leu681	
	Lys457	Asp682	X	Lys457	Asp682	X	Arg401	Glu717		Glu524	Lys758	
	Gln516	Arg811		Glu528	Lys758	X	Arg401	Phe718		Asn548	Arg811	
	Arg521	Arg756		Glu552	Arg807	X	Val425	Glu654		Pro651	Pro653	
				Thr652	Gln656		Lys457	Asp682	X			
							Gln516	Gly748				
							Gln516	Gly748				
							Gln516	Val750				
							Glu517	Arg906	X			
							Glu524	Arg756				
							Glu524	Pro757				
							Glu528	Lys758				
							Asn548	Gly748				
							Asn548	Glu749				
							Asn548	Arg811				
							Glu552	Arg807	X			
	24 Contacts			26 Contacts			37 Contacts			32 Contacts		

RecA1, RecA2 — Helicase Core (HC)

WH, HB, OB — C-Terminal domains (CTD)

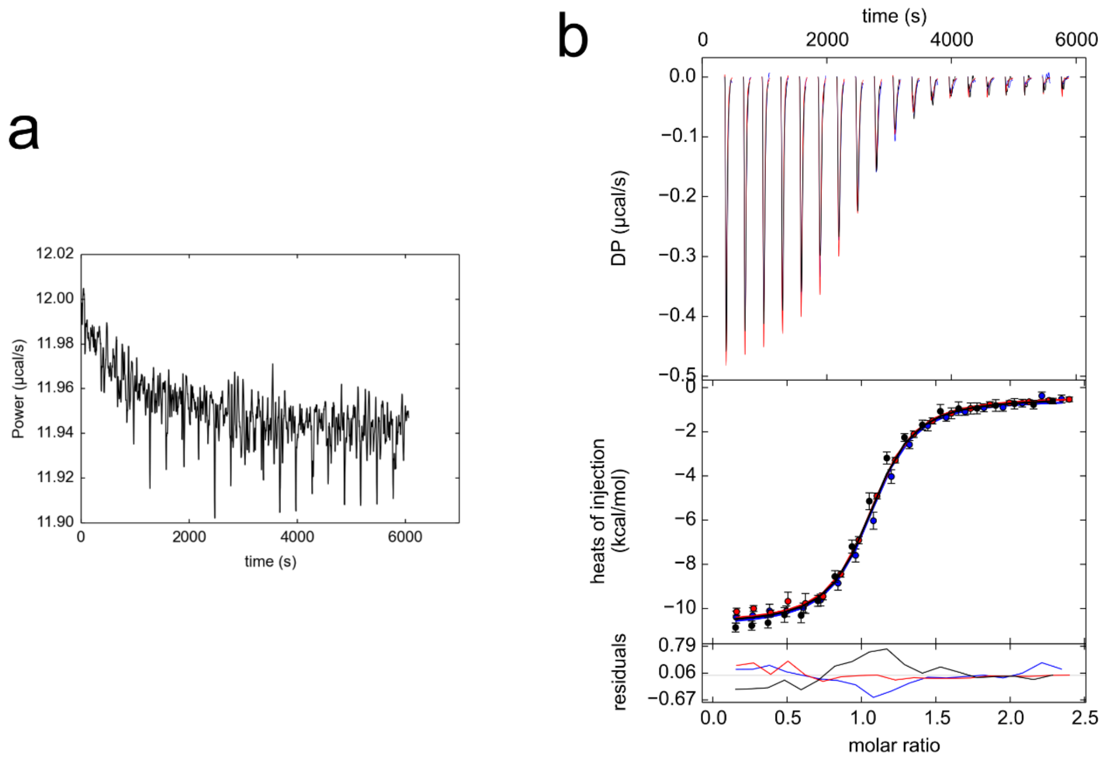

Figure S1 Isothermal titration calorimetry of Prp2 with AMPPCP and ADP. (a) The thermogram of the titration of AMPPCP to Prp2 did not result in thermic signals suited for integration, indicating there is no binding. (b) The binding of ADP was assessed and a dissociation constant (K_d) of 179 nM was determined (n= 1.01, ΔH= -10.63 kcal/mol, ΔS= -5.06 cal/mol*K). Individual triplicates are depicted in red, blue and black. In the upper panel the thermograms are displayed, the middle panel shows the individual fittings with the correspondent residuals in the lower panel.

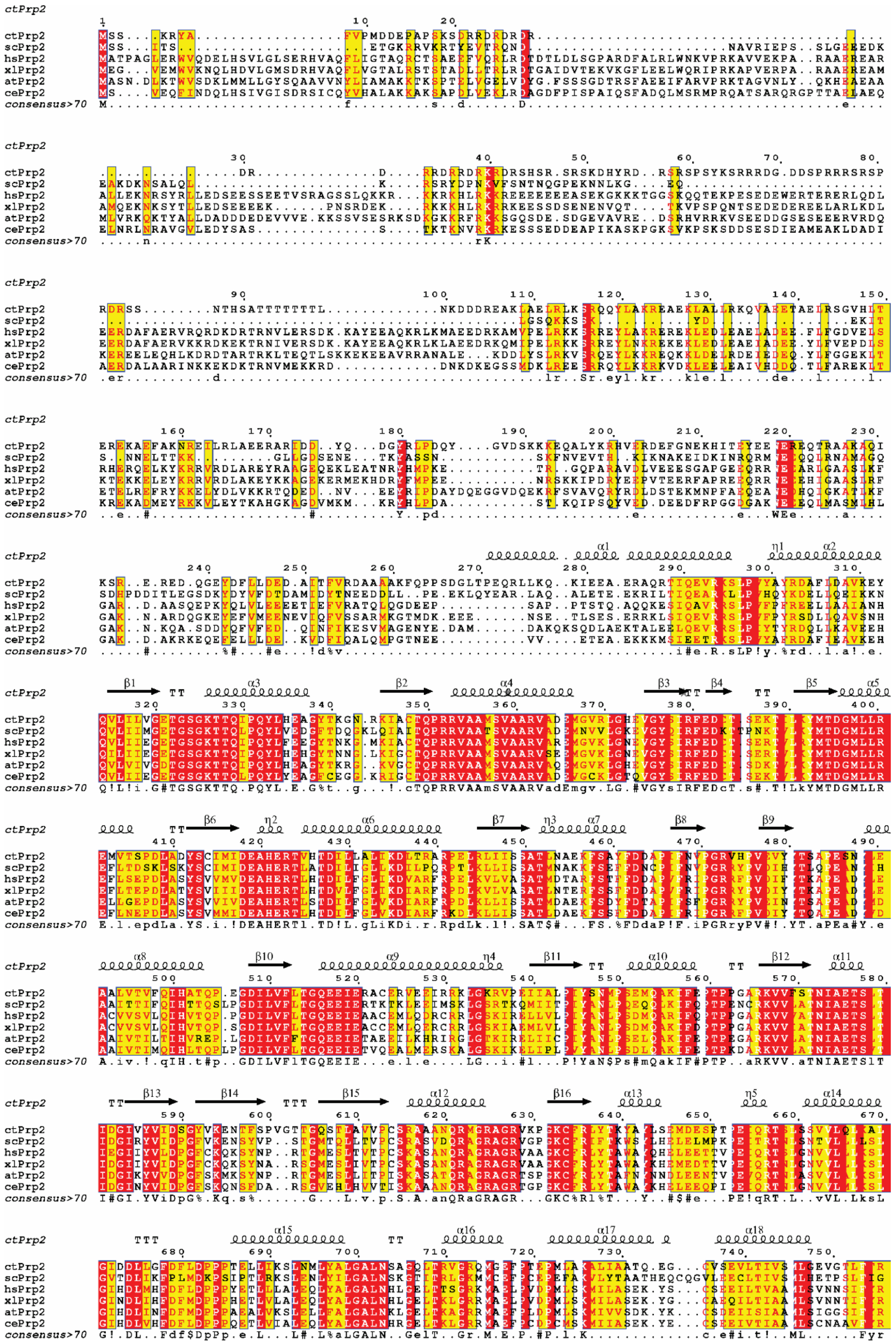
ctPrp2
scPrp2
hsPrp2
xlPrp2
atPrp2
cePrp2
consensus>70

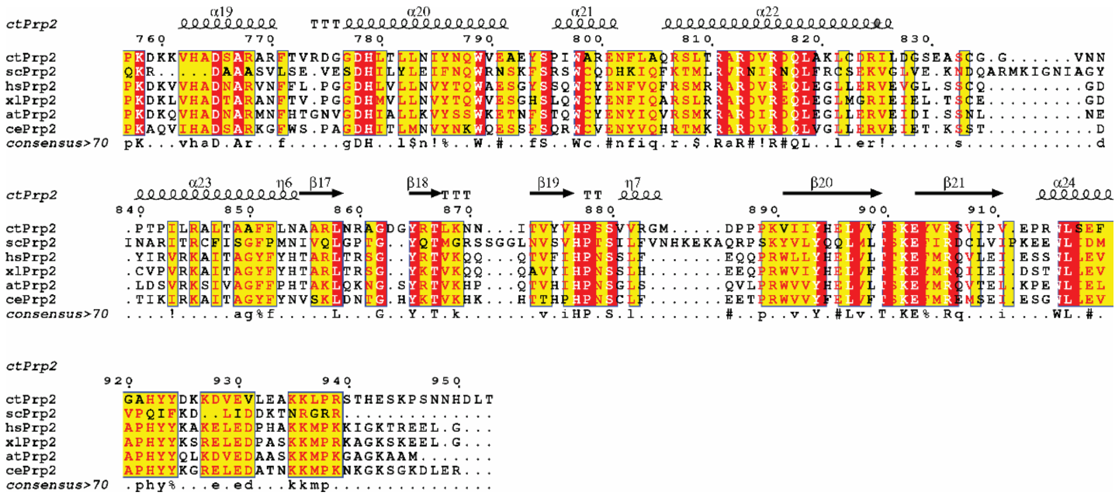

Figure S2 Sequence alignment of Prp2 from *Chaetomium thermophilum* (*ct*), *Saccharomyces cerevisiae* (*sc*), *Homo sapiens* (*hs*), *Xenopus laevis* (*xl*), *Arabidopsis thaliana* (*at*) and *Caenorhabditis elegans* (*ce*). Secondary structure elements from CF2 are displayed on top of the corresponding sequence.

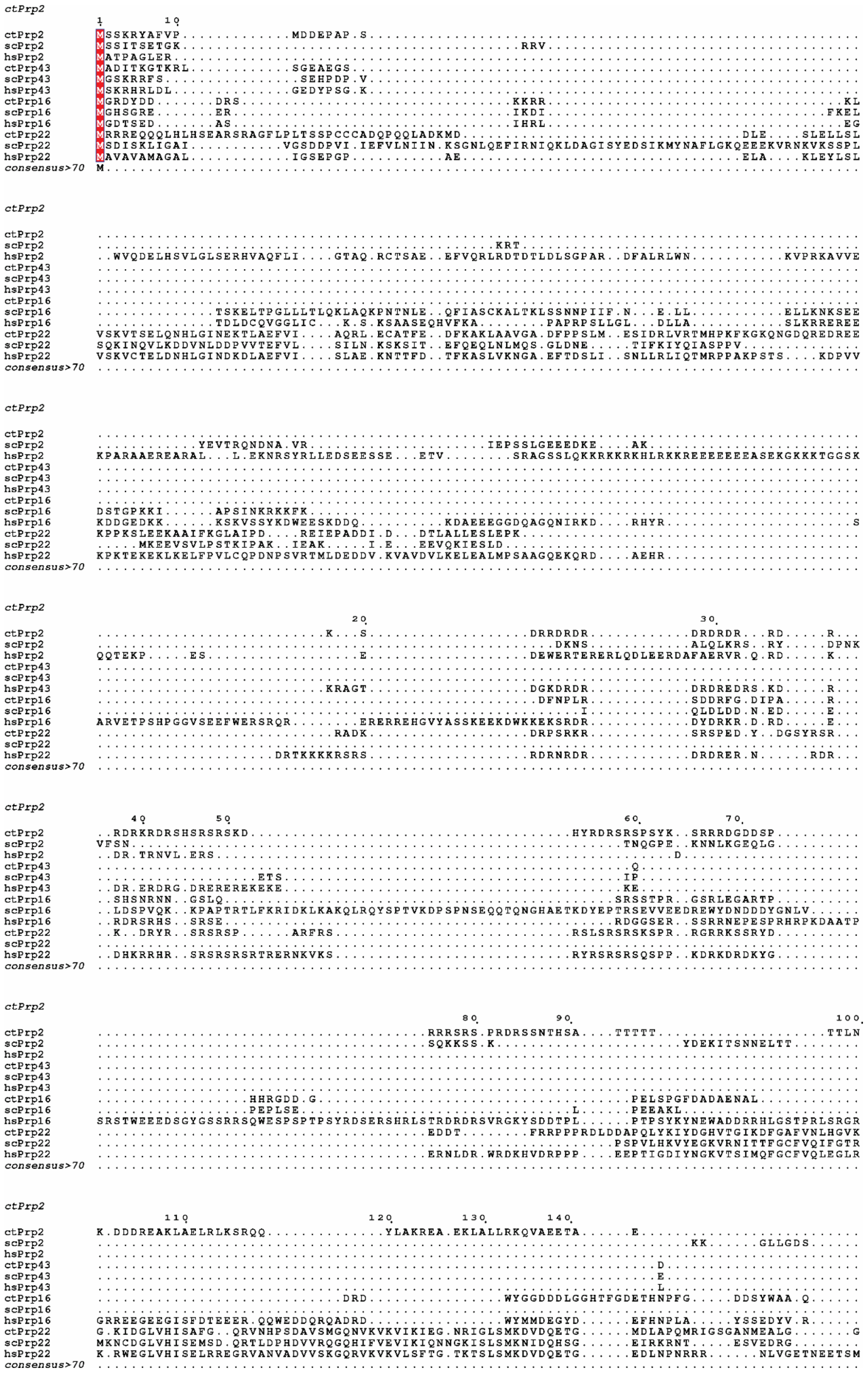

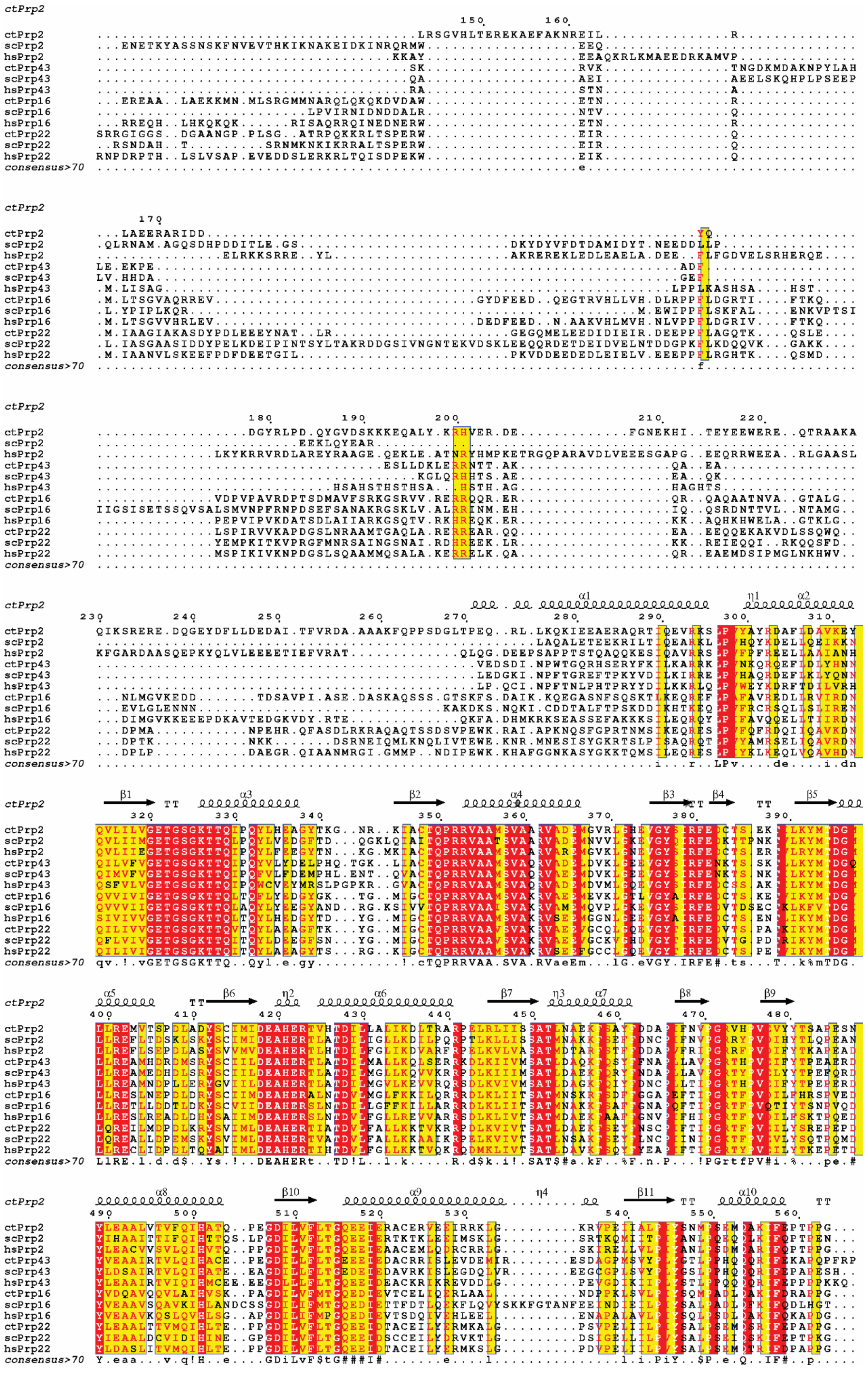

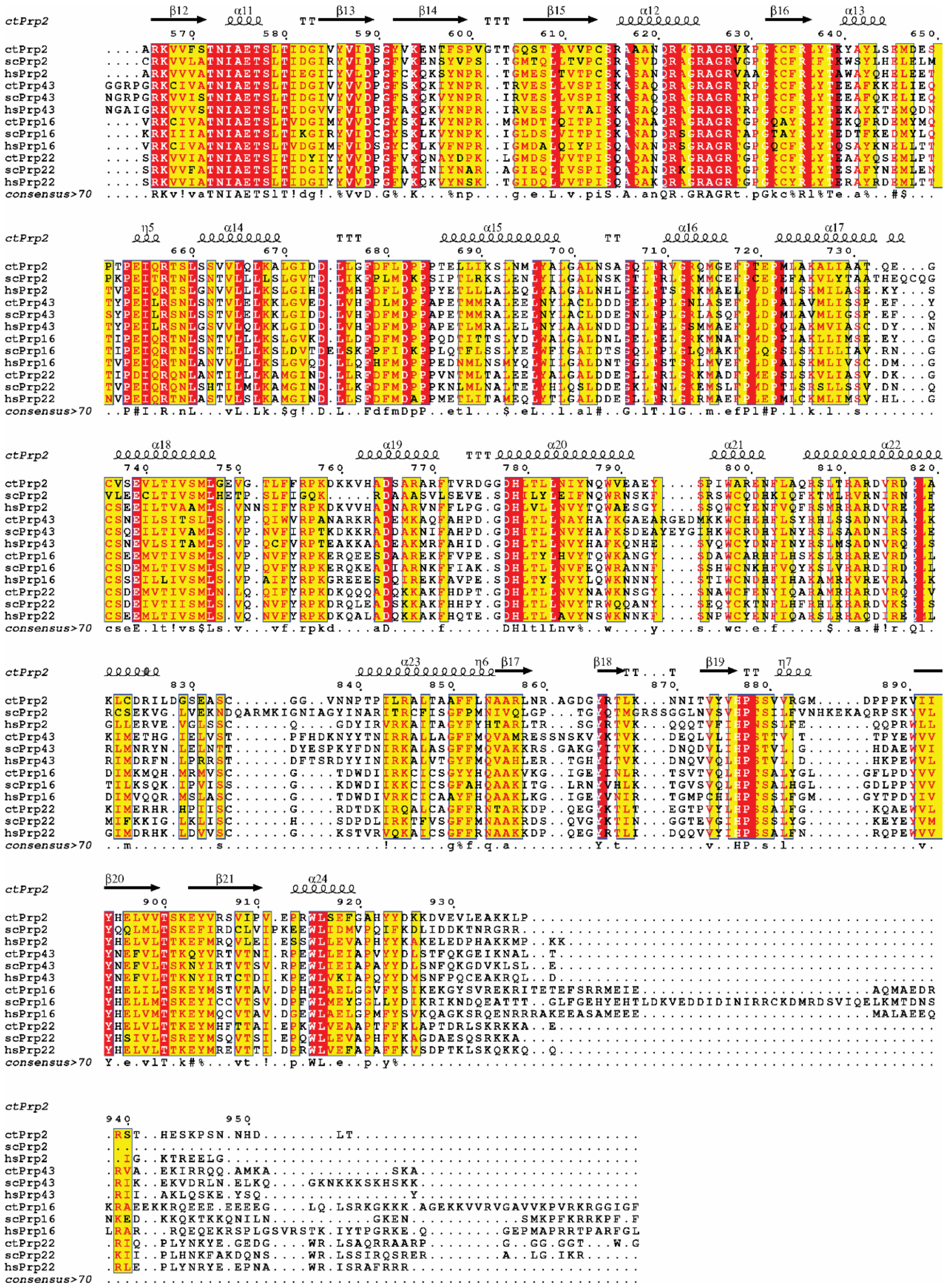

Figure S3 Sequence alignment of Prp2, Prp43, Prp16 and Prp22 from *Chaetomium thermophilum* (*ct*), *Saccharomyces cerevisiae* (*sc*) and *Homo sapiens* (*hs*). Secondary structure elements from CF2 are displayed on top of the corresponding sequence.

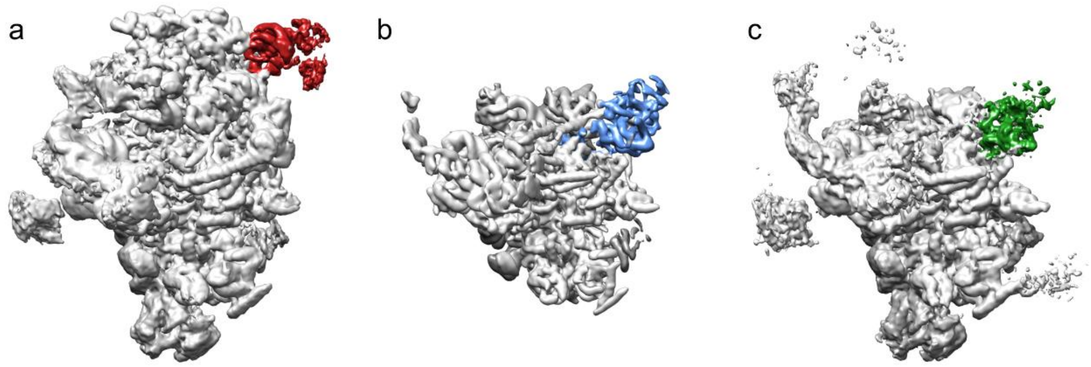

Figure S4 Position of DEAH-box helicases in the spliceosome. The cryo-EM maps of the *Saccharomyces cerevisiae* B^{act} complex (a, Yan *et al.*, 2016), *Saccharomyces cerevisiae* post-catalytic P complex (b, Liu *et al.*, 2017) and *Saccharomyces cerevisiae* spliceosome complex remodelled for exon ligation (c, Fica *et al.*, 2017) superimposed and are shown in grey and DEAH-box helicases of each complex are depicted in red (Prp2), blue (Prp22) and green (Prp16), respectively. All DEAH-box helicases are found in the periphery of their respective spliceosomal complex.

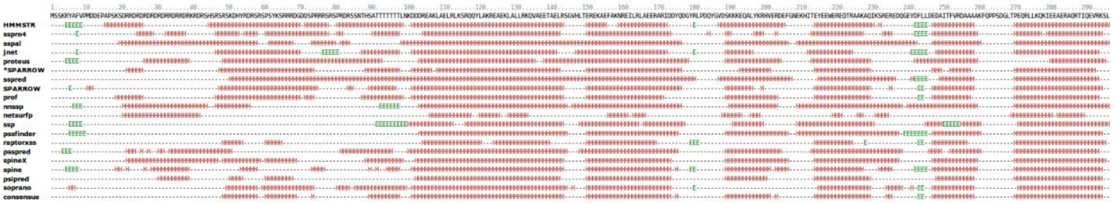

Figure S5 Secondary structure prediction of N-terminal extension of *Chaetomium thermophilum* Prp2. The secondary structure prediction was performed using the GeneSilico metaserver (Kurowski & Bujnicki, 2003). Used prediction server for the metasearch are stated the left followed by the corresponding prediction on the right. Residues predicted to be part of a helical secondary structure are highlighted by “H” whereas residues predicted to be part of beta strands are displayed as “E”.
